# Basil (*Ocimum basilicum*) to Alleviate Anxiety in Patients With Major Depressive Disorder: A Randomized Placebo‐Controlled Clinical Trial

**DOI:** 10.1002/brb3.70994

**Published:** 2025-11-14

**Authors:** Mahva Talaei, Kiarash Zare, Yussef Hashemi, Amir Hesam Pahlevani, Bahareh Fakhraei, Foroogh Namjooyan, Mohammad Hashem Hashempur, Amin Kouhpaye, Sayed Hamdollah Mosavat

**Affiliations:** ^1^ Student Research Committee Fasa University of Medical Sciences Fasa Iran; ^2^ Research Center for Psychiatry and Behavior Science Shiraz University of Medical Sciences Shiraz Iran; ^3^ Department of Psychiatry, Shariati Hospital Fasa University of Medical Sciences Fasa Iran; ^4^ Research Center for Traditional Medicine and History of Medicine, Department of Persian Medicine, School of Medicine Shiraz University of Medical Sciences Shiraz Iran; ^5^ Department of Pharmacology, School of Medicine Fasa University of Medical Sciences Fasa Iran

**Keywords:** anxiety, basil, depression, integrative medicine, *Ocimum basilicum*, traditional Persian medicine

## Abstract

**Background:**

Major depressive disorder (MDD) is a widespread global mood disorder, often accompanied by anxiety as a common symptom. This study assessed the effectiveness of basil (*Ocimum basilicum*) syrup in alleviating anxiety in patients with depression.

**Methods:**

A randomized single‐blinded clinical trial was conducted at Dastgheib Psychiatric Clinic, Fasa University of Medical Sciences. Sixty participants were randomly assigned to receive either basil syrup or a placebo for 4 weeks. Anxiety and depression were assessed using the Hamilton Anxiety Rating Scale (HAM‐A) and Beck Depression Inventory (BDI), respectively, before and after the intervention.

**Results:**

The basil group showed significant reductions in HAM‐A and BDI scores compared to the placebo group (*p* value < 0.05).

**Conclusion:**

Basil syrup could reduce anxiety in patients with MDD. However, further studies with a larger sample size and longer follow‐up period are necessary.

**Trial Registration:**

IRCT20230318057752N1

## Graphical abstract

In a randomized placebo‐controlled trial, basil (*Ocimum basilicum*) syrup significantly reduced anxiety and depression scores in patients with major depressive disorder. Findings suggest basil extract as a safe, well‐tolerated complementary therapy alongside standard antidepressant treatment.

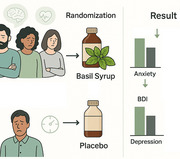

## 1 Introduction

Major depressive disorder (MDD), the most common mood disorder worldwide, is recognized as an increasing social burden and recently has been reported as a significant cause of mortality (Li et al. 2020). Depression can affect thoughts, mood, and physical health and can be associated with numerous symptoms such as low mood, fatigue, sadness, insomnia, anhedonia, and cognitive impairment symptoms (Li et al. 2020; Schulz and Arora 2015). Many factors can affect the prevalence of MDD, including culture, socioeconomic status, age, gender, and marital status (Pramesona and Taneepanichskul 2018; Tuithof et al. 2018). In 2016, about 350 million people were depressed worldwide (Summergrad 2016). The prevalence of MDD in Iran was reported by two systematic reviews, done in 2010 and 2019. Both studies reported a total point prevalence of 4.1%, and both reported that the incidence of MDD was 1.95 times more in women than men (Gharraee et al. 2019; Sadeghirad et al. 2010).

MDD often occurs simultaneously with other psychiatric disorders, including anxiety disorder (59.2%), impulse control disorder (30%), and substance abuse disorder (24%) (Schulz and Arora 2015). Anxiety disorders are the most common associated disorders in depressed patients. They encompass a collection of disorders distinguished by excessive fear, anxiety, or avoiding specific internal or external stimuli. Anxiety disorders are reported as one of the most significant global health challenges, often exerting profound impairments on occupational, social, and various functional aspects of individuals’ lives (Gustavsson et al. 2011; Schulz and Arora 2015).

Only less than 20% of people facing anxiety disorders are treated adequately (Kasteenpohja et al. 2016; Roberge et al. 2015). If these disorders are not treated, they will become chronic. Cognitive behavioral therapy (CBT) is empirically the best type of psychological treatment. Antidepressants are usually the first line of pharmacotherapy, and among antidepressants, selective serotonin reuptake inhibitors (SSRIs) and selective serotonin‐norepinephrine reuptake inhibitors (SNRIs) are mostly used (Craske and Stein 2016). Antidepressants are highly potent medications that can result in serious interactions with a wide range of substances (Marks 2023). Sexual dysfunction is among the most common adverse effects of SSRIs and SNRIs, along with weight gain, gastrointestinal disturbances, and sleep changes (Hutters and Giraldi 2022; Marks 2023; Piazza et al. 2023; Pukall 2023; Sartin‐Tarm et al. 2022; Rothmore 2020; Shelton 2019; Wang et al. 2022).

Such side effects and limitations of common treatment methods have increased the tendency to use complementary and alternative medicine (Mosavat et al. 2023; Vincent and Furnham 1996). According to WHO, about 80% of people living in developing countries have used complementary and alternative medicine (Silva 2015). Herbal medicine is a popular form of traditional medicine, because of not only its biomedical benefits but also cultural beliefs in many parts of the world (Ganesan and Xu 2017; Ghorat et al. 2024). Nowadays researchers still keep looking for novel treatments hidden in the formulas and prescriptions of traditional medical systems (Alyasin et al. 2020; Medina‐Franco et al. 2013; Meysami et al. 2021).

*Ocimum basilicum*, commonly known as basil, is a plant rich in bioactive phytochemicals that offer a variety of therapeutic benefits. This medicinal plant is both inexpensive and widely accessible and has been used for thousands of years (Zhakipbekov et al. 2024). Basil contains bioactive compounds such as linalool, eugenol, and rosmarinic acid, which may exert anxiolytic and antidepressant effects via modulation of gamma‐aminobutyric acid (GABA)ergic transmission, antioxidative and anti‑inflammatory pathways, and enhancement of brain‑derived neurotrophic factor (BDNF) levels (Dhama et al. 2023; Seyed et al. 2021). In traditional medicine, basil has been used to treat many diseases including anxiety, diabetes, osteoarthritis, cardiovascular diseases, headaches, and seizures (Askari et al. 2024; Bora et al. 2011; Umar et al. 2010).

Several studies have examined basil's antianxiety and sedative effects (Gradinariu et al. 2015; Miraj and Kiani 2016; Rabbani et al. 2015). In murine models, basil extract at doses of 200–400 mg/kg reduced anxiety‐like behavior in the elevated plus maze and open‐field tests, and decreased immobility time in forced swim and tail suspension tests, indicating antidepressant potential (Ali et al. 2017; Gradinariu et al. 2015). Although some trials on animal models have been done to discover the anxiolytic and antidepressant effects of basil (Ayuob et al. 2017; Gradinariu et al. 2015; Mostafapour et al. 2018; Rabbani et al. 2015; Sentari et al. 2019; Suryani et al. 2019), only a few pieces of evidence are available on the efficacy of such treatment in humans. This study aimed to evaluate the effect of basil extract on anxiety symptoms in patients with MDD.

## 2 Materials and Methods

### 2.1 Trial Design

This study is a parallel‐design, two‐arm, randomized single‐blind placebo‐controlled clinical trial. In this study, the efficacy of the hydroalcoholic extract of basil leaves in reducing the anxiety level of patients suffering from MDD who are treated with sertraline was assessed.

### 2.2 Participants

Eligible participants were men and women aged 18–65 years diagnosed with MDD according to DSM‐5 criteria. Participants were required to have concurrent symptoms of anxiety, as evidenced by a Hamilton questionnaire score of at least 18, and must not have initiated any new antianxiety medications within the past month. Exclusion criteria included individuals with chronic or significant medical conditions, current use of medications, or substance use disorders, patients who were pregnant or breastfeeding, and patients whose medication had changed during the study.

Diagnosis of MDD was confirmed according to DSM‐5 criteria through a structured clinical interview performed by a board‐certified psychiatrist at the recruitment site. Participants also needed to meet the minimum severity threshold on standardized rating scales, specifically a Beck Depression Inventory (BDI) score corresponding to at least moderate depression and a Hamilton Anxiety Rating Scale (HAM‐A) score ≥ 18, ensuring both depression and clinically relevant anxiety were present at baseline.

### 2.3 Intervention

The study enrolled 60 individuals from a psychiatric clinic, all diagnosed with MDD and experiencing anxiety, after obtaining informed written consent. At the onset, each patient completed the BDI and the HAM‐A questionnaire, along with providing demographic details. Sertraline at a dosage of 50–100 mg/day was given to both groups of the study as standard treatment prescribed by a psychiatrist. Patients in the treatment group were prescribed one 5 mg of basil syrup nightly before sleep. Patients in the control group received 5 mg of placebo syrup nightly before sleep, with the medication appearing identical in shape and packaging to maintain blinding. This treatment regimen was followed for 4 weeks. Participants were informed about the study's aim, procedures, duration, anonymity of their data, and their right to withdraw at any time, without mentioning the expected comparative effect of basil.

All participants were explained that this study was a clinical trial in which they could use herbal syrup to reduce anxiety in addition to standard antidepressant medication. We also told all of them that the syrup they received could be a drug or a placebo.

After 1 week, patients were contacted via phone to monitor for any medication‐related complications and to ensure treatment adherence. Four weeks posttreatment initiation, patients underwent a follow‐up visit where the BDI and the HAM‐A questionnaires were readministered.

The choice of dose and treatment length was based on a synthesis of traditional Persian medicine recommendations, pharmacognosy data, and existing safety/efficacy literatures. Pilot human studies and preclinical data suggested that basil extracts at 1000–1200 mg/day are effective in reducing anxiety symptoms with minimal adverse effects. Accordingly, a syrup containing 1100 mg hydroalcoholic extract per 5 mL was prescribed as a single nightly dose to enhance compliance and minimize gastrointestinal discomfort. A 4‐week duration was selected as the minimum period typically required to observe herbal anxiolytic effects, while limiting attrition and facilitating standardized monitoring.

### 2.4 Preparation of the Study Drug and Placebo

The active study drug was prepared from dried basil leaves. Initially, the leaves underwent a process where extra stems and roots were removed before being dried in the shade at a temperature of 40°C. Following this, the dried leaves were finely ground using a grinder until they reached a particle size of approximately 2 mm. Subsequently, a 70% hydroalcoholic solution was used in conjunction with a Soxhlet extractor to extract the active ingredients, resulting in a dry hydroalcoholic extract.

To formulate the final syrup, the dry extract was dissolved in distilled water to achieve a thick and uniform solution. This concentrated solution was then added to a base syrup, meticulously calculated to achieve a concentration of 1100 mg/5 mL, and adjusted to the final volume.

In parallel, a placebo syrup was prepared following the standards outlined in the American Pharmacopoeia (Taneja et al. 2023). This involved mixing 85 g of sugar with 100 mL of distilled water. The mixture underwent a gradual heating process up to 100°C until achieving uniformity. In addition, a permissible food‐coloring agent was incorporated to match the appearance of the medicinal syrup, ensuring both syrups were visually indistinguishable.

#### 2.4.1 Determination of Total Polyphenol Content

The total polyphenol content (TPC) of the extracts was measured using a modified Folin–Ciocalteu colorimetric method. Briefly, 10 mg of extract was dissolved in 10 mL methanol, and 0.2 mL of this solution was mixed with 2.5 mL of 10% Folin–Ciocalteu reagent. After 2 min incubation in the dark at room temperature, 2 mL of 7.5% sodium carbonate solution was added. Following 120 min of further incubation, absorbance was recorded at 765 nm using a UV–visible spectrophotometer. A standard calibration curve was prepared with gallic acid (25–200 mg/L, *R* ^2^ = 0.99), and results were expressed as mg gallic acid equivalents per gram of extract (mg GAE/g) (Ahmed et al. 2019; Romano et al. 2022).

### 2.5 Sample Size

The sample size for this clinical trial was determined based on the study by Eskandarzadeh et al. (2021), which reported a significant decrease in Hamilton questionnaire scores from 31.9 ± 9 to 15.4 ± 9, corresponding to a reduction of 15 units with a standard deviation (SD) of 9 units. For this trial, a clinically significant effect size (*d*) of 8 units was considered. To achieve a statistical power of 90% with a confidence level of 95%, and assuming a dropout rate of 10%, a minimum of 27 participants per group was calculated. Therefore, the trial was designed to enroll 30 participants in each group to ensure adequate statistical power and account for potential dropouts.

### 2.6 Randomization and Blinding

Randomization of participants was conducted using a restricted randomization technique to ensure equal distribution across the study groups. This was achieved by preparing 30 white balls and 30 black balls, each representing one of the two study groups (basil syrup or placebo). These balls were sequentially removed from a bag without replacement, and their order was recorded.

Following this, opaque and sealed envelopes containing either a black‐ or white‐coded questionnaire were arranged in the same order as the balls were removed and placed into a box. This box was then stationed at the location where the researcher would meet the study participants. Upon a participant's entry into the study, envelopes were handed out in the predetermined order. Participants received these envelopes and completed the questionnaires in a separate room to maintain blinding.

The medications and placebos were prepared in identical boxes, sealed, and labeled according to the black or white code noted on the participant's questionnaire. This labeling process was handled in another room to prevent any bias. The medications were then distributed to the participants based on the code they had received, ensuring that the participants were unaware of whether they were receiving the active medication or the placebo.

Each phase of the study, including the randomization process, observation of questionnaire color codes, and the delivery of medications, was managed by personnel who were not involved in other aspects of the study. This procedure ensured that the study maintained single‐blind conditions, preserving the integrity of the trial and preventing any bias.

### 2.7 Outcome Measures

The clinical trial assessed two outcome measures: the change in symptom severity of depression, evaluated by the BDI, and the severity of anxiety, evaluated by the HAM‐A. These measures were taken both before the intervention and 4 weeks after the intervention.

### 2.8 Ethical Issues

The trial followed the Declaration of Helsinki (1989 revision) and was also reviewed, approved, and monitored by the Ethics Committee of Fasa University of Medical Sciences (License Number IR.FUMS.REC.1401.251). The trial was registered with the Iranian Registry of Clinical Trials with the following code: IRCT20230318057752N1. All the participants signed an informed consent form before enrollment in the study.

### 2.9 Statistical Methods

All analyses were performed using SPSS Statistics version 15.0 (IBM Corp., Armonk, NY, USA). In addition to absolute scores, delta values (change from baseline) were analyzed to compare intervention effects between groups. Descriptive statistics were calculated for demographic and baseline clinical characteristics, presented as means and SDs for continuous variables and frequencies and percentages for categorical variables.

The primary outcome was the change in HAM‐A and BDI scores from baseline to the end of the intervention. Paired *t*‐tests were used to compare pre‐ and post‐intervention scores within each group. Independent *t*‐tests were employed to compare the mean changes between the basil syrup group and the placebo group. A *p* value of less than 0.05 was considered statistically significant. Paired *t*‐tests were employed to compare pre‐ and post‐intervention scores within each group, and independent‐sample *t*‐tests were used to compare mean change scores (delta values) between groups. This approach was chosen to facilitate direct estimation of effect size for clinical interpretation in a relatively small sample.

## 3 Results

### 3.1 Participant Characteristics

**FIGURE 1:**
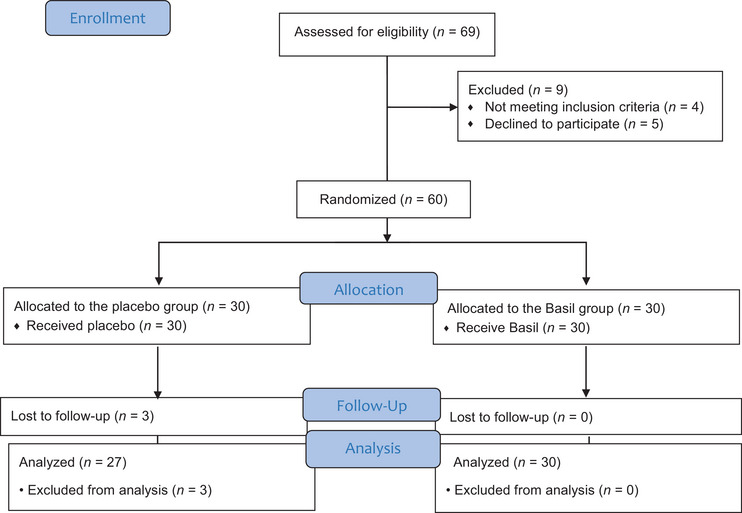
The CONSORT flowchart of the clinical trial.

A total of 60 participants were initially enrolled in the study, with 30 participants allocated to the *O. basilicum* (basil) group and 30 participants to the placebo group. Three participants in the basil group withdrew due to mild gastrointestinal discomfort (*n* = 1) and personal scheduling conflicts unrelated to the intervention (*n* = 2). Figure 1 shows the CONSORT flowchart of the clinical trial.

The average age of participants in the basil group was 34 ± 11 years, while in the placebo group it was 40 ± 12 years. This difference was statistically significant (*p* = 0.046). No statistically significant differences were reported between the groups regarding body mass index (BMI) and gender distribution. Table 1 provides detailed baseline demographic and clinical characteristics.

**TABLE 1:** Baseline demographic and clinical characteristics.

Variable	Basil group (*n* = 27)	Placebo group (*n* = 30)	*p* value
Age (years)	34 ± 11	40 ± 12	0.046
Gender (male/female)	13/14	15/15	0.792
BMI (kg/m^2^)	23.5 ± 3.2	24.1 ± 3.0	0.567

### 3.2 Primary and Secondary Outcomes

As shown in Table 2, before the intervention, the mean BDI scores were 39.44 ± 5.67 in the basil group and 40.3 ± 6.6 in the placebo group. No significant baseline differences in HAM‐A and BDI scores were observed between groups (*p* > 0.4 for both).

**TABLE 2:** Primary and secondary outcome measures.

Outcome measure	Basil group (mean ± SD) (*n* = 27)	Placebo group (mean ± SD) (*n* = 30)	*p* value
BDI score (baseline)	39.44 ± 5.67	40.3 ± 6.6	0.601
BDI score (post‐intervention)	21.04 ± 6.34	29.57 ± 4.62	< 0.001
Change in BDI score	−18.40 ± 6.12	−10.73 ± 5.21	< 0.001
HAM‐A score (baseline)	33.19 ± 4.68	34.53 ± 6.44	0.406
HAM‐A score (post‐intervention)	12.81 ± 4.48	24.8 ± 5.82	< 0.001
Change in HAM‐A score	−20.37 ± 3.40	−9.73 ± 2.80	< 0.001

After the intervention, the scores were 21.04 ± 6.34 in the basil group and 29.57 ± 4.62 in the placebo group. The reduction in BDI scores was significantly greater in the basil group compared to the placebo group (*p* < 0.001).

Before the intervention, the mean HAM‐A scores were 33.19 ± 4.68 in the basil group and 34.53 ± 6.44 in the placebo group. After the intervention, the scores were 12.81 ± 4.48 in the basil group and 24.8 ± 5.82 in the placebo group. The reduction in HAM‐A scores was significantly greater in the basil group compared to the placebo group (*p* < 0.001).

BDI scores of 0–13 indicate minimal depression, 14–19 mild, 20–28 moderate, and 29–63 severe. HAM‐A scores of ≤ 17 indicate mild severity, 18–24 moderate, and ≥ 25 severe. Post‐intervention reductions in the basil group represented a shift from severe to mild anxiety and from severe to moderate depression.

### 3.3 Total Polyphenol Content

The TPC of the hydroalcoholic basil leaf extract in the syrup was calculated based on 1100 mg extract per 5 mL and found to be 15.8 ± 0.8 mg GAE/mL.

### 3.4 Safety

Both the basil and placebo supplements were well‐tolerated, and no serious side effects were observed during the study period. Mild side effects in the basil group included transient gastrointestinal discomfort (*n* = 1) and mild headache (*n* = 2), all resolving without intervention.

## 4 Discussion

Anxiety is a significant health concern that can severely diminish the quality of life. Numerous medical and nonmedical treatments exist for anxiety, including antidepressants, benzodiazepines, buspirone, and CBTs. SSRIs and SNRIs may cause sexual dysfunction, weight gain, gastrointestinal upset, and sleep disturbance, often contributing to early discontinuation (Cuijpers et al. 2013; Lader 2014; Quagliato et al. 2019). In contrast, basil was well‐tolerated, with only mild, transient adverse effects observed. This clinical trial was conducted to investigate the effects of adding *O. basilicum* (basil) syrup to the usual therapeutic regimen of patients dealing with depression and anxiety disorders simultaneously.

In the intervention group, significant reductions in both depression and anxiety levels were observed after receiving the basil treatment, as evidenced by the BDI and HAM‐A scores. Similar significant reductions were noted in the control group, which aligns with previous studies that highlight the therapeutic effects of standard medications such as antidepressants and benzodiazepines on depression and anxiety (Gale and Davidson 2007; Sadock et al. 2017).

Our study findings indicate that while both groups experienced reductions in depression and anxiety levels, a significantly larger reduction in both HAM‐A and BDI scores was observed in the basil group compared with placebo (*p* < 0.001). This suggests that the addition of basil syrup may enhance the efficacy of the standard therapeutic regimen. Previous research has documented the anxiolytic effects of basil and its therapeutic potential in depressed models of rats and mice (Ali et al. 2017).

Studies in animal models (rats and mice) demonstrated that both hydroalcoholic extracts and essential oils of basil significantly increased time spent in open arms of elevated plus maze tests (a standard anxiety measure), indicating anxiolytic effects (Gradinariu et al. 2015; Nemati et al. 2015; Rabbani et al. 2015). These extracts also reduced locomotor activity, reflecting a sedative effect. Essential oil showed stronger effects than the extract at equivalent doses (Gradinariu et al. 2015). A study on rats subjected to chronic restraint stress (an anxiety model) showed that basil hydroalcoholic extract significantly reduced anxiety‐like behavior by increasing entries and time spent in open arms and improving depressive‐like behavior in forced swimming tests (Mostafapour et al. 2018). The antianxiety effects may be attributed to the antioxidative and neuroprotective properties of phenolic, flavonoid, and tannin compounds present in basil (Khan et al. 2023). Though clinical trials on *O. basilicum* specifically for generalized anxiety disorder are limited, a related species, *Ocimum tenuiflorum* (Holy Basil), has clinical evidence suggesting stress reduction and improvement in subjective anxiety measures (Lopresti et al. 2022).

Our study further corroborates these findings, indicating that basil syrup could be a valuable adjunctive treatment for anxiety and depression. The hypothesized mechanisms through which basil exerts its antidepressant and anxiolytic effects include its influence on the central nervous system through the modulation of neurotransmitters. Basil contains several bioactive compounds such as linalool, eugenol, and rosmarinic acid, which are believed to contribute to its therapeutic effects. Linalool, a major component of basil, has been shown to have sedative and anxiolytic effects, potentially by modulating the (GABA)ergic system (Dhama et al. 2023; Qneibi et al. 2024). Eugenol, another key component, may exert anxiolytic effects through its antioxidant properties and by modulating the dopaminergic and adrenergic systems (Goyal et al. 2023). Rosmarinic acid, known for its anti‐inflammatory and antioxidant properties, may also play a role in reducing anxiety and depression by modulating neuroinflammatory pathways and enhancing BDNF levels (Ghasemzadeh Rahbardar and Hosseinzadeh 2020). These mechanisms collectively suggest that basil's bioactive compounds can influence various pathways involved in mood regulation, contributing to its antidepressant and anxiolytic effects (Ayuob et al. 2017; Gradinariu et al. 2015; Miraj and Kiani 2016; Mostafapour et al. 2018; Rabbani et al. 2015; Sentari et al. 2019).

Moreover, the growing interest in the anxiolytic and antidepressant properties of plants is evident from recent research. Numerous plants, including celery seed, lemon balm, ashwagandha root, *Nigella sativa*, ginger, and chamomile, have been investigated and proven to have therapeutic effects on anxiety and depression (El Joumaa and Borjac 2022; Garg et al. 2023; Sabri et al. 2022). This study aligns with the broader trend of exploring and utilizing herbal medicines for mental health treatment.

The promising results from this trial suggest that basil syrup could significantly contribute to the management of anxiety and depression when used in conjunction with standard treatments. The findings support the potential of herbal medicine as a complementary approach, offering a safer and possibly more effective treatment option for patients with anxiety and depression.

### 4.1 Limitations

There are several limitations to consider in this study. The relatively short follow‐up period may not capture long‐term effects of basil syrup on anxiety and depression. The sample size, although adequate for initial findings, may limit the generalizability of the results. Future studies with larger sample sizes and extended follow‐up periods are warranted to validate these findings. Given the frequent comorbidity of depression and anxiety, improvements in one may influence the other, making it difficult to disentangle basil's specific effects. In this study, we attempted to achieve double‐blinding; however, given the potential taste and smell of basil syrup, we are uncertain whether blinding was fully completed. So, a limitation of our study is that, despite identical packaging, the distinct aroma and flavor of basil may have partially unblinded participants, potentially introducing bias.

## 5 Conclusion

This randomized, single‐blind, placebo‐controlled clinical trial found that the use of basil (*O. basilicum*) extract in addition to the standard antidepressant sertraline was more effective than placebo in reducing symptoms of both depression and anxiety in patients with MDD. The basil group demonstrated significantly greater improvements in BDI scores and HAM‐A scores compared to the placebo group after 4 weeks of treatment. The basil syrup was well‐tolerated, with no serious adverse events reported. These findings suggest that basil extract may have therapeutic potential as a complementary treatment option for managing anxiety symptoms in patients with MDD. The low cost, widespread availability, and traditional use of basil make it an attractive natural alternative or adjunct therapy. Further larger‐scale studies are warranted to confirm these results and elucidate the precise mechanisms by which basil exerts its anxiolytic and antidepressant effects.

## Author Contributions

**Mahva Talaei**: Data collection, patient recruitment, project administration, and initial draft preparation. **Amir Hesam Pahlevani**: Data collection, patient assessment, and statistical analysis. **Yussef Hashemi**: Methodology design, psychiatric evaluation, and supervision of patient assessment. **Kiarash Zare**: Data curation, validation, and assistance in manuscript drafting. **Bahareh Fakhraei**: Clinical supervision, participant follow‐up, and review of psychiatric scales. **Amin Kouhpaye**: Conceptualization, study design, pharmacological formulation, statistical analysis, and critical revision of the manuscript. **Seyed Hamdollah Mosavat**: Conceptualization, study supervision, funding acquisition, and manuscript revision. **Foroogh Namjooyan**: Resources, quality control of herbal preparation, and manuscript review. **Mohammad Hashem Hashempur**: Project supervision, methodological validation, and final approval of the manuscript. All authors have read and approved the final manuscript.

## Conflicts of Interest

The authors declare no conflicts of interest.

## Peer Review

The peer review history for this article is available at https://publons.com/publon/10.1002/brb3.70994.

## Data Availability

The data supporting this manuscript's findings are not publicly available due to Ethical restrictions. Data are, however, available from the authors upon reasonable request and with permission of Shiraz University of Sciences.
